# The relationship between alignment, function and loading in total knee replacement: In-vivo analysis of a unique patient population

**DOI:** 10.1016/j.jbiomech.2020.110042

**Published:** 2020-11-09

**Authors:** David Williams, Andrew Metcalfe, June Madete, Gemma Whatling, Peter Kempshall, Mark Forster, Kathleen Lyons, Cathy Holt

**Affiliations:** a Cardiff School of Engineering, College of Physical Sciences and Engineering, Cardiff University, UK; b Warwick Clinical Trials Unit, University of Warwick, Coventry, UK; c Department of Electrical and Electronic Engineering, Kenyatta University, Nairobi, Kenya; d Gloucestershire Hospitals NHS Foundation Trust, Great Western Road, Gloucester, UK; e Cardiff and Vale University Health Board, Cardiff, UK; f University Hospital of Wales, Cardiff, UK

**Keywords:** TKR, Arthroscopy, Frontal plane alignment, Gait analysis, Fluoroscopy, TKR, Total knee replacement, TCWSM, Treatment Centre Weston Super Mare, HKA, Hip-Knee-Ankle angle, PTSA, Posterior Tibial Slope Angle, PROMS, Patient Reported Outcome Measure, KOS, Knee Outcome Survey, GRF, Ground Reaction Force, KAAI, Knee Adduction Angular Impulse, EKAM, External Knee Adduction Moment, AP, Anterior-Posterior, ROM, Range of Motion, PE, Polyethylene

## Abstract

The purpose of this study was to quantify the effect of total knee replacement (TKR) alignment on in-vivo knee function and loading in a unique patient cohort who have been identified as having a high rate of component mal-alignment. Post-TKR (82.4 ± 6.7 months), gait analysis was performed on 25 patients (27 knees), to calculate knee kinematics and kinetics. For a step activity, video fluoroscopic analysis quantified in-vivo implant kinematics. Frontal plane lower-limb alignment was defined by the Hip-Knee-Ankle angle (HKA) measured on long leg static X-rays. Transverse plane component rotation was calculated from computed tomography scans. Sagittal plane alignment was defined by measuring the flexion angle of the femoral component and the posterior tibial slope angle (PTSA). For gait analysis, a more varus HKA correlated with increased peak and dynamic joint kinetics, predicting 47.6% of Knee Adduction Angular Impulse variance. For the step activity, during step-up and single leg loaded, higher PTSA correlated with a posterior shift in medial compartment Anterior-Posterior (AP) translation. During step-down, higher PTSA correlated with reduced lateral compartment AP translation with a posterior shift in AP translation in both compartments. A more varus HKA correlated with a more posterior medial AP translation and inter-component rotation was related to transverse plan range of motion. This in-vivo study found that frontal plane lower-limb alignment had a significant effect on joint forces during gait but had minimal influence on in-vivo implant kinematics for step activity. PTSA was found to influence in-vivo TKR translations and is therefore an important surgical factor.

## 1 Introduction

Total knee replacement (TKR) is a successful operation for treating pain and improving function in end stage osteoarthritis, with 102,177 replacements performed in the United Kingdom (excluding Scotland) in 2017 (National Joint Registry, 2018). Despite its popularity, up to 20% of patients are dissatisfied with their outcome, experiencing ongoing pain and poor function (Bourne et al., 2010, Price et al., 2018, Wylde et al., 2007). The choice and positioning of TKR components relative to the femur and tibia, both surgeon-controlled factors, can influence patient outcomes (Bonner et al., 2011, Czurda et al., 2010). For optimal TKR function (restoration of near normal motion and function), implant components should be correctly aligned either to mechanical frontal plane parameters (Mechanical alignment) or to recreate native knee anatomy (Kinematic alignment) (Bäthis et al., 2004, Sikorski, 2008). It is currently unclear which is optimum.

Frontal plane component alignment, transverse plane component rotation, flexion angle of the femoral component and posterior slope angle of the tibial component are all surgeon-controlled. In-vitro studies have demonstrated the effect of varying frontal plane alignment (D’Lima et al., 2001, Green et al., 2002, Vandekerckhove et al., 2017, Werner et al., 2005).

Few in-vivo studies (Fujito et al., 2018, Harman et al., 2012) have explored these factors. Patient cohorts with significant implant component mal-alignment are relatively rare (Parratte et al., 2010, Sikorski, 2008), thus limiting in-vivo biomechanics investigations to patients with relatively small alignment variations. Thus, a comprehensive analysis of the relationship between component positioning and in-vivo function and loading has not been reported.

In 2004, by way of reducing the nearly three-year waiting list for Welsh TKR surgeries, patients were offered treatment at the NHS Treatment Centre Weston Super Mare (TCWSM), which has since closed. 224 patients (258 knees) were treated at the TCWSM using a Stryker Kinemax TKR prosthesis. This implant has been shown to have a high survival rate of 96.1% after nine years within normal clinical settings (Back et al., 2001, Bannister et al., 2010, Wright et al., 2004), and in one cohort, a 16.3-year survival rate of 84% (Grazette et al., 2018). For the 224 TCWSM patients, the five-year survival rate was 80.6% (Hickey et al., 2012) with surgeon-noted high levels of implant mal-alignment. The cause has not been identified and is not the purpose of this paper (Kempshall et al., 2009).

The current paper presents an important exploratory analysis involving this unique patient population with a single implant design having a high rate of mal-alignment. As a retrospective exploratory study, a group of TCWSM patients underwent gait analysis and video fluoroscopy providing a unique opportunity to investigate the influence of three-dimensional TKR implant alignment on knee kinematic biomechanics, function and loading in-vivo.

The primary hypothesis tested whether frontal plane lower-limb alignment determined by Hip-Knee-Ankle angle (HKA), influences knee biomechanics when measured during level gait using marker-based motion capture.

The secondary hypothesis tested whether surgical alignment, including HKA, transverse plane component rotation, flexion angle of the femur and slope angle of the posterior tibial component, influences in-vivo implant kinematics during a step activity when measured using video fluoroscopy.

## 2 Methods

Participants with the Kinemax (Stryker) cruciate retaining TKR implant treated at the TCWSM were recruited. Approval was granted by the Wales Research Ethics Committee 3 (Ref:10/MRE09/28) for Cardiff and Vale University Health Board and written informed consent obtained from each participant prior to data collection.

All patients received clinical assessment with radiographs 2–3 years post operatively as part of their review. To show the full range of surgical knee alignment, a systematic approach was taken to split the original cohort into quartiles based on the X-Ray measures reported in Kempshall et al. (2009). From the original cohort of 224 patients (254 knees), ten patients were invited from three groups, the first and fourth quartile and a combination of the middle two quartiles. The initial approach was made by the orthopaedic team in charge of their ongoing care at that time.

Exclusion criteria for this study were patients with a hip replacement or severe hip OA in the same leg, evidence of previous extremity trauma or other extra-articular deformity in the same limb, neurological disease or other co-morbidity which affects gait pattern. Evidence of radiographic loosening based on the clinical radiographs was also an exclusion criterion for the study.

From the original 224 patients (254 knees), and with the exclusion criteria applied, 29 patients (34 affected knees) were recruited into this study.

### 2.1 Alignment and surgical parameter analysis

Frontal plane lower-limb alignment was calculated using HKA from the mechanical axes of the femur and the tibia from long leg radiographs (Cooke et al., 2007). Additional surgical alignment measures were transverse plane femoral and tibial component rotation, posterior slope angle of the tibial component (commonly termed Posterior Tibial Slope Angle, PTSA) and flexion angle of the femur component post-surgery.

**Fig. 1:**
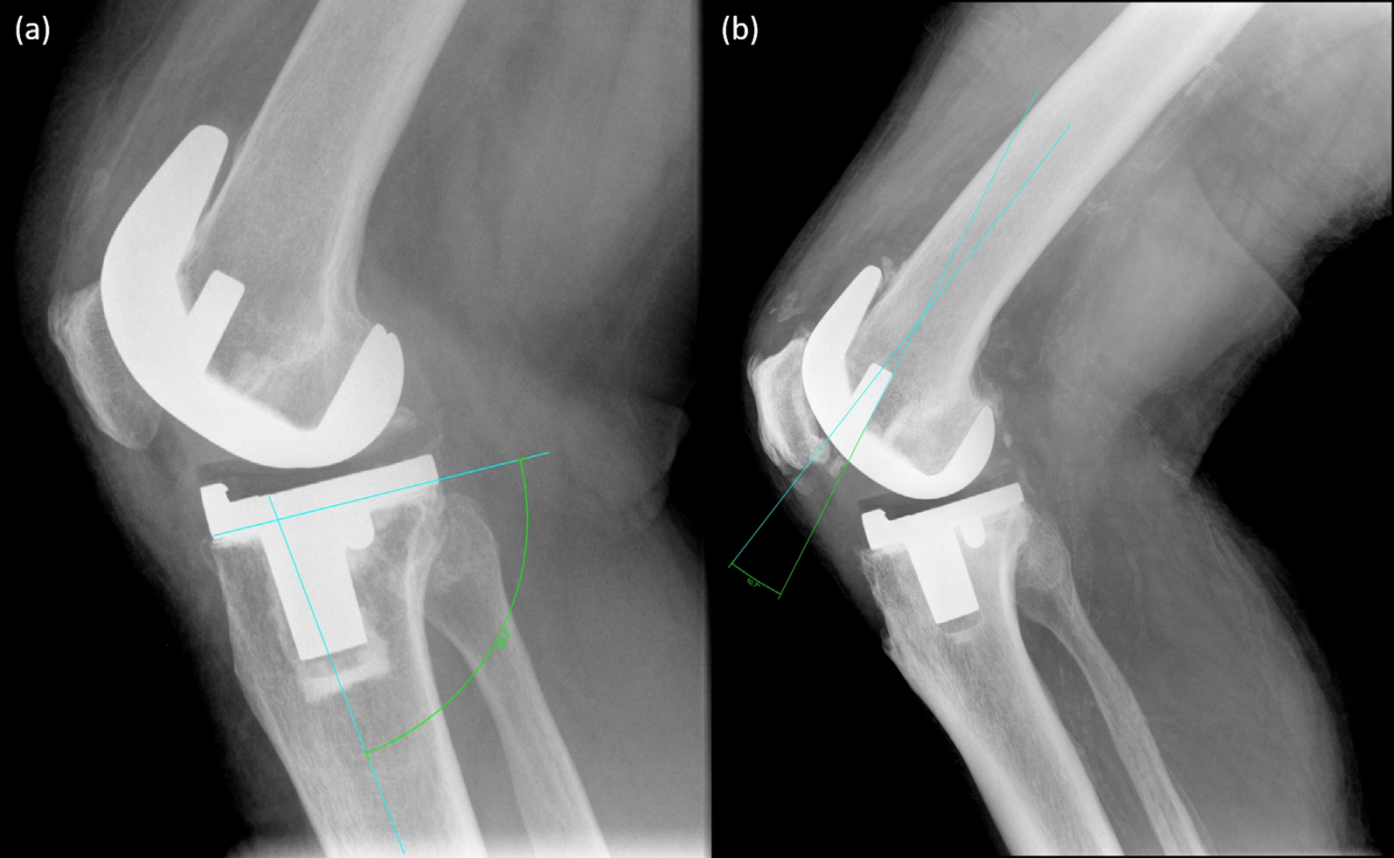
(a) Posterior tibial slope angle was measured from clinical plane X-Rays at the intersection of a line drawn across the tibial plateau and a line drawn down the middle of the tibial shaft joining two centre-points at 10 and 20 cm; (b) The flexion angle of the femur was defined as the angle between the back of the anterior flange of the femoral component, and a line drawn along the middle of the femoral shaft on the lateral knee x-ray.

The transverse plane component rotation was determined from limited slice CT scans (GE Discovery) of the affected knee based on the Perth protocol (Chauhan et al., 2004). To reduce radiation dose hip/ankle scans were omitted and adaptive statistical iterative reconstruction with a slice thickness of 4 mm used. One experienced consultant surgeon carried out radiological measurements developed for this study. Transverse plane femoral component rotation was defined as the angle between the most prominent point of both femoral epicondyles and the femoral implant (measured using the posterior margin of the anterior flange of the femoral component). Transverse plane tibial component rotation was defined as the angle between the tibial posterior cortical line in the first bony slice under the implant and the tibial pegs. PTSA and flexion angle of the femur were defined on clinical X-rays as described in Fig. 1.

### 2.2 Gait analysis and patient reported outcome measures

Three-dimensional marker-based motion analysis was performed on all participants 82.4 ± 6.7 months post- TKR surgery. Pre-analysis, participants were asked to complete Patient Reported Outcome Measures (PROMS) including the Oxford Knee Score (OKS) (Dawson et al., 1998), Knee Outcome Survey (KOS) (Irrgang et al., 1998) and a visual analogue pain score. Gait analysis was performed with a 8-camera system (60 Hz, ProReflex, Qualisys, Sweden), synchronised with two force plates (600 mm × 400 mm, 1080 Hz, Bertec Corporation, Ohio, USA) built into a walkway. 22 retroreflective markers were attached to bony landmarks and rigid clusters positioned laterally on the thigh and shank, based upon the CAST marker set (Benedetti et al., 1998, Cappozzo et al., 1996).

Subjects walked at self-selected speed for a minimum of 6 successful trials. Where participants struggled to complete or data issues were identified, a minimum of 3 trials were used for analysis.

Visual 3D (C-Motion Inc., Maryland, USA) was used to compute knee kinematics and kinetics with joint angles calculated using the Cardan/Euler X, Y, Z convention (Grood and Suntay, 1983, Wu et al., 2002).

External knee moments were resolved in the local coordinate system of the distal segment; suggested to be closest to representing internal knee loading (Brandon and Deluzio, 2011, Schache et al., 2008, Zhao et al., 2007). Ground reaction forces (GRF) were normalised to bodyweight and joint moments normalised to the percentage bodyweight times height.

Following recommendations taken from McClelland et al. (2007) the most commonly measured kinematic and kinetic metrics for analysing TKR function were calculated from gait analysis (Table 1). The Knee Adduction Angular Impulse (KAAI) was calculated during stance phase to investigate the magnitude and duration of the external knee adduction moment (EKAM) (Levinger et al., 2013). EKAM, representing the moment of the GRF vector acting around the knee joint centre, has been shown to be a surrogate measure of medial compartment contact force (Andriacchi, 1994, Kutzner et al., 2013, Zhao et al., 2007). KAAI, the integral of the EKAM curve, provides useful measures of dynamic loading by combining both magnitude and direction into one variable.

**Table 1:** Kinematic and kinetic outputs from gait analysis.

	Parameters
Kinematic (°)	Sagittal Plane range of motion
	Transverse Plane range of motion
	Frontal Plane range of motion
	Angle flexion at initial Contact
	Flexion range of motion during stance Phase
	Maximum Flexion angle during Swing
	Maximum Flexion angle during Stance
	Minimum Flexion angle during Stance
	Maximum abduction angle (Positive value)
	Maximum adduction angle (Negative value)

Ground Reaction Force [%BW]	Maximum of the first peak in the vertical Ground Reaction Force
	Minimum after first peak in the vertical Ground Reaction Force
	Maximum of the Second peak in the vertical Ground Reaction Force
	Maximum of the Anterior-Posterior Ground Reaction Force
	Minimum of the Anterior-Posterior Ground Reaction Force
	Maximum Medial Ground Reaction Force
	Maximum Lateral Ground Reaction Force
	Maximum Flexion Moment

External Knee Moments [%BW.h]	Maximum Extension Moment
	Maximum adduction moment
	Maximum abduction moment
	Maximum Internal Rotation Moment
	Maximum External Rotation Moment
	Maximum Extension Moment at Initial Contact

	Knee Adduction Angular Impulse (%BW.h.seconds)

### 2.3 Fluoroscopic analysis of a step activity

**Fig. 2:**
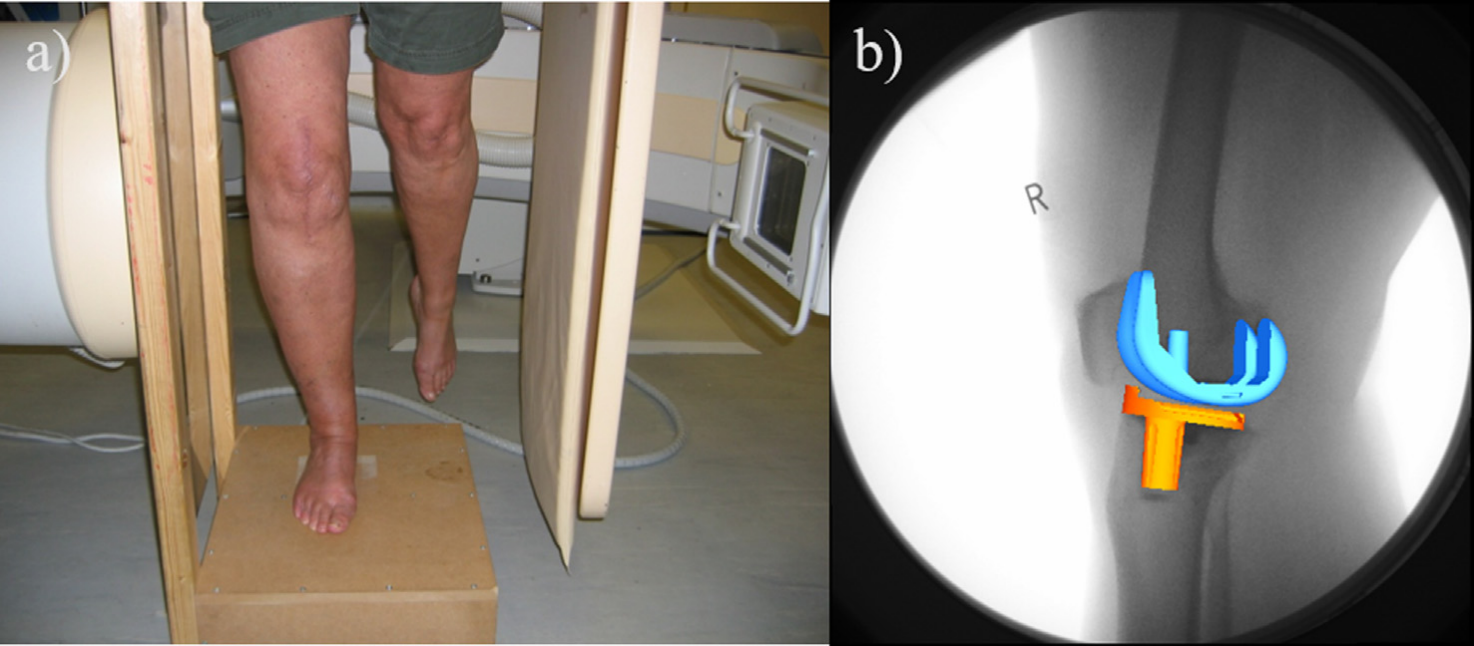
Fluoroscopy data collection and 3D to 2D image registration. a) Patient volunteer performing the step activity while video fluoroscopy is captured. b) Example processed fluoroscopy framed processed using JointTrack showing 3D computer models of the femoral and tibial component aligned with the 2D X-ray frame.

Knee motion was observed using video fluoroscopy (MD Eleva; Philips) as subjects performed a single leg step-up-step-down activity on a 16 cm step (Fig. 2a). Starting with their ipsilateral foot on the step, subjects were instructed to step-up to full knee extension, then reverse direction back to the starting position whilst avoiding contralateral limb swing into view of the fluoroscope. Thirty 2D fluoroscopic images per second were acquired and digitally converted into DICOM images. To calculate geometric distortion and optical geometry of the fluoroscope, a calibration object consisting of two Perspex sheets with embedded stainless steel beads was imaged (Banks and Hodge, 1996).

An open source package, JointTrack (Mu, 2007), was used to determine the 3D location and orientation of the TKR components from the fluoroscopic images. CAD models were provided by the company (Stryker, USA). Anatomical/implant coordinate systems were applied using Rhinoceros 4 (Robert McNeel & Associates, USA) following the approach defined by (Banks and Hodge, 1996). Combined manual and shape matching techniques (Fig. 2b), produced an accuracy of 0.5 mm and 1° (Banks and Hodge, 1996).

Cardan/Euler angles were adopted to calculate kinematics from the 3D positions of the femur and tibia components (Tupling and Pierrynowski, 1987). Medial and lateral compartment contact points were calculated for each frame using a nearest neighbour algorithm. Anterior-posterior (AP) translation on the tibial tray was determined based on the AP contact points (Guan et al., 2017). Stair activity was split into step-up, single leg loaded and step-down.

### 2.4 Statistical analysis

All statistical analyses were performed using R v3.6.2 (R Core Team, 2019) and figures produced using ggplot2 (Wickham, 2016).

For the primary analysis, investigative correlations using Spearman’s Rank correlation coefficient compared clinical measures (HKA/PROMs), with gait analysis outputs (kinematic/kinetic) obtained from motion capture. For correlations found to have high statistical significance (R> ± 0.70 and P < 0.001), a linear regression model was evaluated by splitting the dataset randomly into training and testing sets (80/20).

For the secondary analysis, a Pearson’s or Spearman’s Rank correlation coefficient (parametric and nonparametric respectively), compared fluoroscopic step-up-step-down activity outputs with surgical measures (transverse plane tibial, femoral and intercomponent rotations; flexion angle of the femoral components; PTSA and HKA). All analyses were based on a Shapiro-Wilks normality test. The patient cohort size was relatively small, thus multiple testing correction was not performed, and all analysis considered exploratory.

## 3 Results

### 3.1 Participants

From the 29 recruited patients (34 affected knees), three measurements were affected by motion capture technical issues and two were affected by fluoroscopic image issues (5 and 2 affected knees respectively). The final patient cohort (Table 2) including linked fluoroscopy, motion analysis and radiological measurements (Table 3) was 25 (27 affected knees).

**Table 2:** Demographics and clinical characteristics for patient volunteers.

	Sex (F/M)	Age (y)	BMI (kg/m^2^)	Mass (kg)	Height (m)	Oxford Knee Score	VAS for pain (%)	Knee Outcome Survey (%)
Overall Mean (SD)	12M 15F	75 (7)	31.1 (6.2)	83.2 (19.6)	1.63 (0.08)	34.8 (10.2)	16.7 (22.2)	56 (16.4)

BMI - Body Mass Index.

VAS - Visual Analogue Scale for pain.

**Table 3:** Radiographic measurements of patient cohort.

	Hip Knee Ankle Angle (°)	Femoral Component Rotation (°)	Tibial Component Rotation (°)	Intercomponent rotation (°)	Flexion angle of Femoral Component (°)	Posterior Tibial Slope Angle (°)
Overall Mean (Range)	1.2 (-9.5 to 10)	−1.3 (-6.0 to 5.7)	4.4 (-8 to 10.3)	0.1 (-8.8 to 8.0)	3.4 (-0.7 to 9.9)	4.9 (-3 to 11)

### 3.2 Gait analysis of level walking

**Fig. 3:**
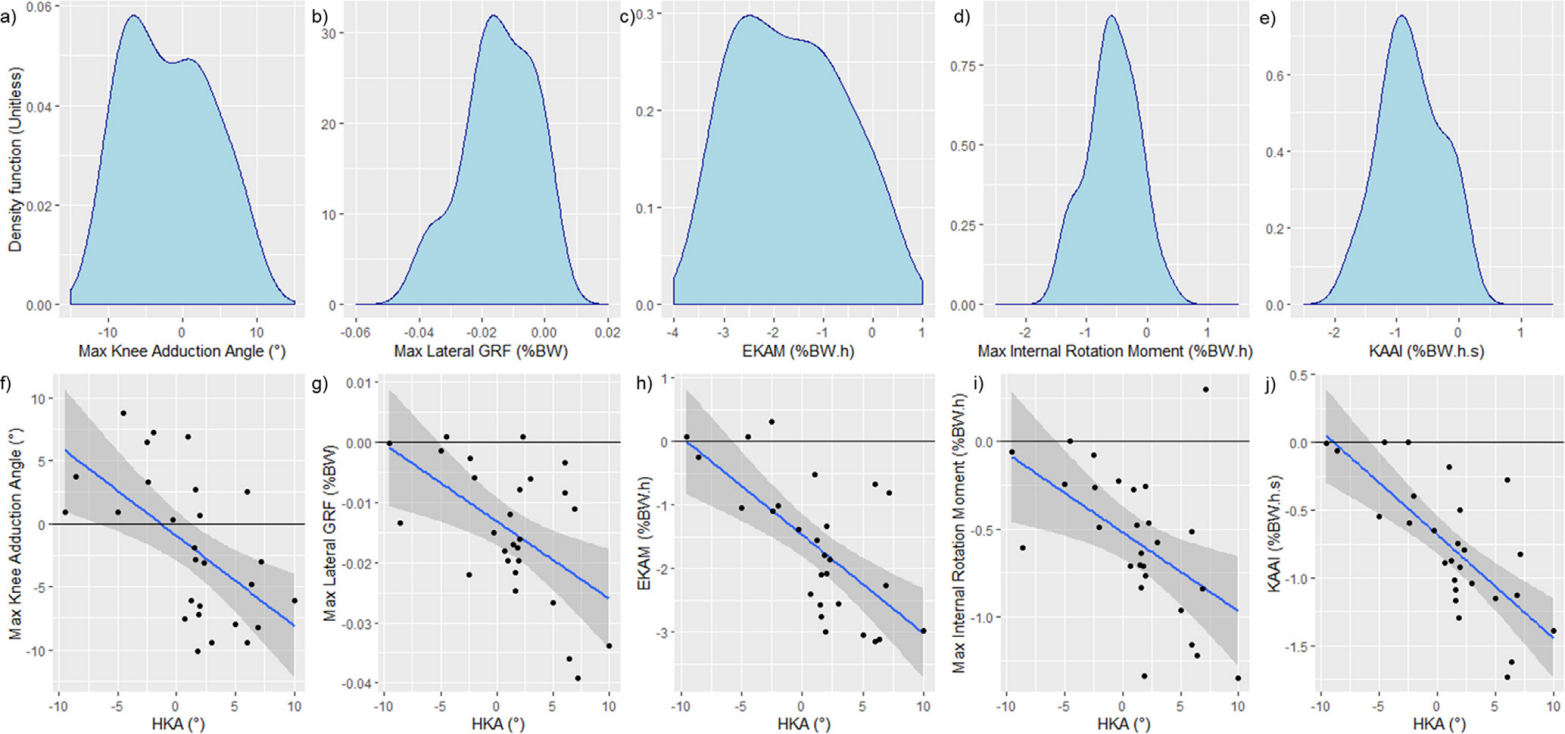
Density (a-e) and scatter plots (f-j) of significant relationships between HKA and Gait analysis outputs.

From the investigative Spearman’s Rank correlation coefficient analysis, during gait, HKA correlated with KAAI (*r* = -0.727, *P* < 0.0001, Fig. 3e,j), maximum EKAM (r = -0.667, *P* = 0.0001, Fig. 3c,h), maximum knee adduction angle (*r* = -0.578, *P* = 0.0002, Fig. 3a,f), maximum lateral GRF (*r =* -0.534, *P* = 0.004, Fig. 3b,g), and maximum knee internal rotation moment (*r =* -0.505, *P* = 0.007, Fig. 3d,i). No correlations were found for PROMS.

The relationship between HKA and KAAI (having *r* ≥ -0.7), was explored using linear regression. Visual inspection of individual variable density (Fig. 3e) and box plots indicated a normal distribution with no significant outliers. HKA predicted KAAI (*F*(1,19) = 17.23, *P =* 0.0005), with the model explaining 47.6% of variance in KAAI (*R^2^* = 0.476, *β* = -0.07211, Intercept = -0.685). Model performance had 79.7% correlation prediction accuracy evaluated by comparing predicted with training set values.

### 3.3 Fluoroscopy analysis of a step activity

Table 4 shows correlations for intercomponent rotation with transverse plane knee range of motion (ROM) (*r* = -0.423, *P* = 0.036), and flexion angle of the femoral component with maximum knee flexion angle (*r* = -0.473, *P* = 0.013). A higher PTSA, was found to correlate with a reduction in maximum flexion angle (*r =* -0.577, *P =* 0.002), and flexion ROM (*r =* -0.432, *P =* 0.031).

**Table 4:** Surgical alignments against fluoroscopy kinematic outputs.

†Spearman’s correlation coefficient (non-parametric data)

Statistically significant,* *p*,0.5 ** *p* < 0.01

AP - Anterior-Posterior translation

ROM - Range of Motion

Higher PTSA correlated with a posterior shift in medial compartment AP translation during step-up (Max Anterior [*r =* -0.448, *P =* 0.019], Max Posterior, [*r =* -0.482, *P =* 0.011]) and single leg loaded (Max Anterior [*r =* -0.558, *P* = 0.002], Max Posterior [*r =* -0.535, *P =* 0.004]).

During step-down, higher PTSA correlated with reduced lateral compartment AP translation (*r =* -0.483, *P =* 0.011), and a posterior shift in AP translation in both compartments (Medial Max Anterior [*r =* -0.463, *P =* 0.003], Max Posterior, [*r =* -0.556, *P =* 0.003]; Lateral Max Anterior [*r =* -0.572, *P =* 0.002]). A more varus HKA correlated with a more posterior medial compartment AP translation (*r* = 0.452, *P =* 0.018).

No correlations were found for transverse plane femoral or tibial component rotations, apart from inter-component rotation, with any fluoroscopy outputs (Table 4).

Table 5 shows a cross-correlation table between all surgical clinical measurements. PTSA was found to have a weak correlation with the transverse plane tibial component rotation (*r =* -0.389, *P =* 0.045), and a moderate correlation with HKA angle (*r =* -0.502, *P =* 0.008). Transverse plane tibial component rotation was found to have a strong correlation with intercomponent rotation (*r =* -0.693, *P <* 0.001).

**Table 5:** Surgical clinical alignment cross-correlation.

†Spearman’s correlation coefficient (non-parametric data)

Statistically significant,* *p*,0.5 ** *p* < 0.01

## 4 Discussion

This study revealed that for this unique cohort of patients a more varus HKA angle was associated with increased peak and dynamic frontal plane loading during gait, indicated by the peak EKAM and KAAI. Furthermore, PTSA was found to have the greatest influence on implant kinematics during the step-up-step-down activity with HKA being shown to have limited influence on implant kinematics. This may have implications in the ongoing debate on kinematic vs mechanical alignment.

Increased EKAM and KAAI suggest a varus aligned TKR experiences higher frontal plane moments acting over a longer duration of stance phase. Ten patients were found to have dynamic loading similar to levels observed in patients with moderate medial OA (indicated by KAAI > -1.0), suggested by (Levinger et al., 2013, Thorp et al., 2006). Two valgus-aligned patients had an overall external abduction knee moment (Fig. 3j) with no KAAI. Several studies exploring frontal plane alignment and loading during gait analysis have suggested that EKAM is a surrogate dynamic measure of knee joint loading (Andriacchi, 1994, Hunt et al., 2006). One potential consequence of a high peak EKAM in an implanted knee is increased wear. Several studies have found increased medial wear for varus aligned implants compared with more neutral alignment (Collier et al., 2007, D’Lima et al., 2001, Matsuda et al., 1999, Werner et al., 2005). Polyethylene (PE) wear was a more common reason for implant revision 15 years ago when the implant in this study was being used. For contemporary implants, revision due to PE wear is less prevalent due to improved biomaterials (Sharkey et al., 2013). In an instrumented knee study, (Kutzner et al., 2017) found that HKA angle correlated with increased medial implant loading during walking. The regression analysis performed in the current study provides further evidence that clinically measured HKA angle is predictive of frontal plane dynamic knee loading; this strong relationship further highlighting the importance of appropriate surgical alignment.

The secondary analysis suggests that during step-up-step-down, as the tibial slope increases, contact on the medial compartment tibial plateau shifts posteriorly, but the range of AP translation remains the same. A shallow (low) PTSA is therefore potentially linked to a more anterior contact location, with a reduced extensor mechanism lever arm and increased patellofemoral joint loading. The decrease in in-vivo maximum flexion and flexion ROM found with increasing PTSA during step-up-step-down is converse to findings reported in the literature.

One other in-vivo study examining the influence of PTSA on knee kinematics using fluoroscopic analysis found no posterior displacement of the femoral condyles and increased flexion ROM during a deep knee bend activity for a high geometric conformity implant (Fujito et al., 2018). It is important to note however that the activity performed was considerably different to that of the current study, thus limiting comparison of kinematics and loading. Several in-vitro studies (Chambers et al., 2016, In et al., 2009, Okazaki et al., 2014), have reported that increasing the PTSA increases flexion ROM. One in-vitro study (Stoddard et al., 2013), found that the Stryker Kinemax design had greater anterior-drawer laxity compared to the natural knee suggesting susceptibility to changes in AP contact location compared with other more conforming implants. Other in-vitro studies (Giffin et al., 2004, Liu-Barba et al., 2007, Meyer and Haut, 2008, Meyer and Haut, 2005), have found that increasing the PTSA under various loading and flexion conditions caused increased anterior translations of the tibia (posterior femoral displacement) with associated increased ACL tension. An in-silico study predicted that increasing PTSA up to 10° led to increased tibial anterior translation and ACL tension, with PCL slackening (Shelburne et al., 2011).

The surgical malalignment in the cohort reported here was not limited to one clinical plane which could provide an explanation for the difference in flexion angles. Singh et al. (2013) looked at the effect of retaining the original pre-surgery anatomical alignment, analysis of pre-post-operative tibial slope differences suggested that recreation of the anatomical tibial slope appears to improve maximum flexion after posterior-stabilised TKR, provided coronal alignment has been restored. Differences in findings could also be related to the defined fluoroscopy activity. The volunteer starts in maximum knee flexion, influenced by lower leg length because of the fixed step height.

In the secondary analysis, intercomponent rotation was found to correlate negatively with knee internal-external rotation ROM during the step-up-step-down activity where tibial rotation was identified as the primary determinant of intercomponent rotation. Increased intercomponent rotation has been shown to link to increased knee pain (Bell et al., 2014), and patellofemoral complications (Berger et al., 1998), and current accepted recommendations are to avoid any mal-rotation (Gromov et al., 2014). Acceptable tolerances for surgical transverse component rotational alignment are not well defined at present. The consequences of mal-rotation could be related to altered knee rotational ROM where increasing external intercomponent mal-rotation constrains internal-external implant kinematics, thus adding stress to ligaments and potential for patellar mal-tracking.

In comparison to much of the literature, the data collected in this study was captured in-vivo providing insight into how TKR is performing for a realistic activity. However, the patients here had potentially more than one type of mal-alignment and PTSA was only moderately associated with HKA and weakly associated with tibial rotation. This highlights the fact that the results of surgical alignment error would most likely be evident in all three planes (rather than one). Despite this, the associations found with the PTSA from the fluoroscopy analysis of a step-up-step-down activity suggest that the main surgical influence on in-vivo implant translations may be sagittal tibial positioning rather than frontal plane positioning.

The results of the fluoroscopic analysis show that HKA angle had minimal influence on in-vivo implant kinematics, except for the posterior position of the medial contact points during step-down. The authors are unaware of any other study that has looked at the influence of frontal plane lower-limb alignment on in-vivo implant kinematics calculated using dynamic fluoroscopic analysis for a step-up-step-down activity. This important finding suggests that neither accidental nor intentional alignment of a TKR off the neutral axis will necessarily influence in-vivo implant kinematics or loaded TKR behaviour. Whilst the authors are unable to comment on local tissue stresses, which may change with altered alignment, this finding indicates that frontal plane lower-limb alignment, as a metric, should not be the primary focus for surgeons or researchers aiming to improve TKR biomechanics.

The findings of this study may have implications in the ongoing debate on kinematic and mechanical alignment, especially as frontal plane alignment seems to have an influence on loading but not in-vivo kinematics. However, there is no pre-surgery alignment data available for this cohort so whether these patients received mechanical alignment or whether the position was closer to an unintentional kinematic alignment is not known. When considering the debate between mechanical and kinematic alignment, it is important to appreciate that all three planes need to be considered and the sole focus should not be on the frontal plane. It is important that the sagittal and transverse planes are appropriately addressed in any attempt to improve in-vivo biomechanics with better alignment.

Some limitations must be considered when interpreting the results of this study. Single plane fluoroscopy has lower precision along the axis of the X-Ray beam, therefore medial-lateral translations were not included in this analysis. The fluoroscope used was limited in frame rate and had minor blurring of some images during acquisition. However, the activity was performed slowly enough for these limitations to not affect data processing. This study, by its nature, is an analysis of an unexpected event and was performed on a patient cohort who were identified retrospectively, therefore any pre-operative clinical measurements were not available for comparison. This includes pre-operative PROMS and pre-arthroplasty radiographic alignment. Although the study has examined post-operative alignment and function only, rather than change introduced by surgery, it has identified important factors to consider when undertaking future prospective studies. Although cohort size is limited, no other reported study has carried out such a comprehensive biomechanical analysis. The large amount of resulting data has proven invaluable and while limited to a hypothesis generating exercise, it has identified important factors for future studies and provides a comprehensive assessment of which surgical factors are of greatest importance for optimising function in knee arthroplasty.

In keeping with other studies, it has revealed that during gait, frontal plane lower-limb alignment (HKA angle), has a significant influence on TKR biomechanical function and loading. Patients with a more varus alignment had an increased frontal plane peak moment and angular impulse, indicating high levels of medial TKR loading over a longer time period. However, during the step activity recorded using dynamic fluoroscopy, the influence of frontal plane lower-limb alignment (HKA angle), on in-vivo implant kinematics was minimal, except for altered posterior location of the medial compartment contact points during step-down.

Based on this study, the recommendation remains to continue the most widely used current practice, aiming for a neutral frontal plane alignment, although further research into the relationship between pre- and post-operative alignment and in-vivo biomechanics across all three planes is needed to refine understanding further. The findings will contribute to the ongoing debate regarding different forms of alignment. Tibial positioning (especially PTSA) and inter-component rotation appear to be critical in providing satisfactory in-vivo implant kinematics and this must be investigated further.

## Declaration of Competing Interest

The authors declare that they have no known competing financial interests or personal relationships that could have appeared to influence the work reported in this paper.
